# Enhancement of Orthodontic Tooth Movement by Local Administration of Biofunctional Molecules: A Comprehensive Systematic Review

**DOI:** 10.3390/pharmaceutics16080984

**Published:** 2024-07-25

**Authors:** Cristina Dora Ciobotaru, Dana Feștilă, Elena Dinte, Alexandrina Muntean, Bianca Adina Boșca, Anca Ionel, Aranka Ilea

**Affiliations:** 1 Department of Oral Rehabilitation, Faculty of Dentistry, “Iuliu Hațieganu” University of Medicine and Pharmacy, 400012 Cluj-Napoca, Romania; ciobotaru_cristina_dora@elearn.umfcluj.ro (C.D.C.); ionel.anca@umfcluj.ro (A.I.); 2 Department of Orthodontics, Faculty of Dentistry, “Iuliu Hațieganu” University of Medicine and Pharmacy, 400012 Cluj-Napoca, Romania; 3 Department of Pharmaceutical Technology and Biopharmaceutics, Faculty of Pharmacy, “Iuliu Hațieganu” University of Medicine and Pharmacy, 400012 Cluj-Napoca, Romania; edinte@umfcluj.ro; 4 Department of Paediatric Dentistry, Faculty of Dentistry, “Iuliu Hațieganu” University of Medicine and Pharmacy, 400012 Cluj-Napoca, Romania; alexandrina.muntean@umfcluj.ro; 5 Department of Histology, Faculty of Medicine, “Iuliu Hațieganu” University of Medicine and Pharmacy, 400012 Cluj-Napoca, Romania; bosca@umfcluj.ro

**Keywords:** orthodontic tooth movement, tooth movement acceleration, biomolecules, drugs, local administration, topical formulation

## Abstract

Enhancement of orthodontic tooth movement (OTM) through local administration of biofunctional molecules has become increasingly significant, particularly for adult patients seeking esthetic and functional improvements. This comprehensive systematic review analyzes the efficacy of various biofunctional molecules in modulating OTM, focusing on the method of administration and its feasibility, especially considering the potential for topical application. A search across multiple databases yielded 36 original articles of experimental human and animal OTM models, which examined biofunctional molecules capable of interfering with the biochemical reactions that cause tooth movement during orthodontic therapy, accelerating the OTM rate through their influence on bone metabolism (Calcitriol, Prostaglandins, Recombinant human Relaxin, RANKL and RANKL expression plasmid, growth factors, PTH, osteocalcin, vitamin C and E, biocompatible reduced graphene oxide, exogenous thyroxine, sclerostin protein, a specific EP4 agonist (ONO-AE1-329), carrageenan, and herbal extracts). The results indicated a variable efficacy in accelerating OTM, with Calcitriol, Prostaglandins (PGE1 and PGE2), RANKL, growth factors, and PTH, among others, showing promising outcomes. PGE1, PGE2, and Calcitriol experiments had statistically significant outcomes in both human and animal studies and, while other molecules underwent only animal testing, they could be validated in the future for human use. Notably, only one of the animal studies explored topical administration, which also suggests a future research direction. This review concluded that while certain biofunctional molecules demonstrated potential for OTM enhancement, the evidence is not definitive. The development of suitable topical formulations for human use could offer a patient-friendly alternative to injections, emphasizing comfort and cost-effectiveness. Future research should focus on overcoming current methodological limitations and advancing translational research to confirm these biomolecules’ efficacy and safety in clinical orthodontic practice.

## 1. Introduction

In contemporary clinical practice, there is a growing demand for esthetic improvements, including those related to dentofacial features, among both young and adult populations. This increasing interest has led to a wider demographic seeking quicker orthodontic treatments, a particularly acute need among adults [1,2]. To address the call for faster treatment, orthodontic professionals are increasingly turning to interventions capable of accelerating tooth movement such as mechanical-physical methods (gene therapy, low-level laser application, electric current or vibration devices), surgical interventions (corticotomies, dentoalveolar distraction, corticocision, piezoincizion, micro-osteoperforations) or “chemical applications” through the use of biofunctional molecules, known to significantly speed up the process of tooth movement [3,4,5]. This makes treatments not only faster but also more aligned with the high expectations of patients for rapid and satisfactory outcomes.

Orthodontic Tooth Movement (OTM) is a complex biological process involving cellular and molecular responses to mechanical forces applied to teeth [6]. When mechanical stress is applied to the periodontium, an aseptic inflammatory reaction occurs, disrupting the homeostasis of the periodontal ligament (PDL) and causing areas of ischemia and vasodilation [7]. Thus, the PDL mediates the processes of alveolar bone apposition and resorption by its components (cells, extracellular matrix, and nerve terminals), resulting in the release of numerous substances including neurotransmitters, cytokines, growth factors, metabolites, and others [8]. It has already been demonstrated in animal models that functional biomolecules such as cytokines, prostaglandins, leukotrienes, immunoglobulins, growth factors, vitamin D and its metabolites, proteins, and thyroid and parathyroid hormones have the ability to interfere with the biochemical reactions that cause tooth movement during orthodontic therapy, accelerating the OTM rate [5,8,9].

This could be of particular interest in clinical practice, since the duration of orthodontic treatment varies depending on malocclusion type and treatment methods [10]. On average, treatment duration is between 24.9 months [11] and 32.2 months for adult patients [12]; however, certain conditions, e.g., the need for extraction therapy, impacted maxillary canines, or the orthodontic closure of edentulous first mandibular molar spaces, are known to increase the treatment time. Shorter treatments are beneficial to the patient, whereas longer treatments carry a higher risk of dental and periodontal issues, and also increase associated costs. In the recent decades, a growing interest in accelerating OTM has been observed. A particular focus was on the local administration of biomolecules, a method seen as a promising approach for shortening the treatment time [13,14]. This focus is driven by a gap in the current literature regarding the comparative efficacy of different administration methods and their practical implications in clinical orthodontics.

This systematic review aims to investigate the outcomes, actions, and efficiency of various biomolecules in enhancing OTM. Special emphasis is placed on the method of administration, particularly exploring the feasibility and effectiveness of topical application. By examining various biomolecules, biosubstances, and their potential applications, this systematic review seeks to provide insights that could influence future clinical practices and guide further research in the field.

In order to facilitate a follow-up on the characteristics sought in the aforementioned biofunctional molecules, this systematic review aims to offer a comprehensive understanding of their role in enhancing OTM (human and animal model), with a detailed analysis of administration methods. This systematic review aspires to bridge the knowledge gap and investigate findings that could have significant implications for orthodontic treatment strategies, potentially leading to more efficient and time-friendly approaches, especially in adult patients.

## 2. Materials and Methods

This systematic review has been registered with the Open Science Framework (OSF) to ensure transparency and standardization in systematic reviews. The registration information, including the protocol and any amendments, can be found at https://doi.org/10.17605/OSF.IO/F6ZY4 (accessed on 19 June 2024).

**Eligibility Criteria**: The PICOS framework was used (population, intervention, comparison, outcome, and study design) in order to assess the eligibility criteria for the studies included in the review. Population comprised healthy human participants or healthy animal subjects undergoing active OTM. Interventions included the local administration of biofunctional molecules or biosubstances with potential to accelerate the rate of tooth movement while comparison involved orthodontic treatment against a placebo, different dosages of the evaluated substances, or no intervention. Outcomes were measured quantitatively and qualitatively, focusing on the rate of tooth movement and the health of surrounding tissues. The study design criteria were restricted to experimental, prospective, controlled trials, both randomized and non-randomized. This systematic review was conducted without time limitations and articles were considered without any language restrictions, allowing for an inclusive and global understanding of the topic.

In more detail, the inclusion and exclusion criteria are presented in Table 1.

**Information Sources and Search Strategy:** A meticulous search was performed across eight electronic databases and two electronic registers up to October 2023. The databases included Embase, Science Direct, ProQUEST, Scopus, Web of Science Core Collection, Medline, Cochrane Central Register, and ClinicalTrials.gov. In addition, a manual search was conducted through the references of included studies to ensure the inclusion of grey literature.

No restrictions were applied regarding language, year of publication, or status of studies during the literature search.

The search combinations of keywords and MeSH terms used can be found in Table 2.

**Selection Process and Data Collection Process**: References were saved using JabRef Bibliography Management v.5 [15], a reference management tool, which was also used for automatic deduplication. The initial data extraction was performed by a single author and subsequently verified by the entire author committee, ensuring accuracy through cross-validation. Discrepancies were resolved through consensus, fortified by professional discourse, until a unanimous decision was reached.

**Risk of Bias Assessment and Reporting of Biases**: For assessing the quality of the selected studies, the Cochrane Risk of Bias tool [16] was used in order to summarize the overall Risk of Bias (RoB) for each human trial, while the risk of bias of the animal studies was assessed using SYRCLE’s risk of bias tool for animal studies. Despite being anticipated, the insufficient number of papers made analysis for publication bias, small-study effects, or exploratory subgroup analyses unfeasible. Two authors independently assessed all publications included in the analysis.

**Data Synthesis and Analysis**: Data extraction from human and animal studies encompassed study design, demographic details, sample size, study duration, intervention specifics (including the substance used and its administration pathway), applied force magnitude, tooth movement type, administration frequency, dosage, and outcome measures. Despite the robust data collection, the heterogeneity of the data precluded a meta-analysis.

**Funding and Ethics Statement**: This research was funded by ”Iuliu Hațieganu” University of Medicine and Pharmacy, Cluj-Napoca, PCD 2023–2024, grant number 646/6/11 of January 2024. Given the nature of a systematic literature review, no ethical approval was required.

## 3. Results

**Figure 1:**
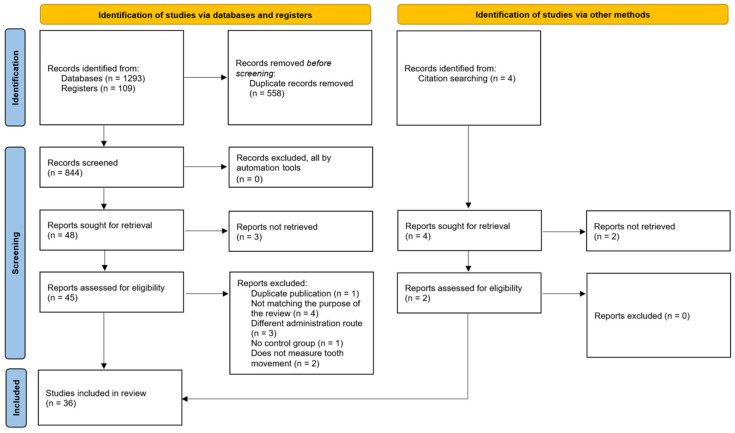
PRISMA 2020 flowchart.

The conducted screening process is graphically expressed in the Prisma 2020 Diagram (Figure 1). An initial yield of 1406 records underwent a systematic screening process, with duplicates removed via the JabRef Bibliography Management tool v.5. The 848 remaining studies after the automatic deduplication process went through a rigorous selection process that led to the exclusion of 795 records based on title and abstract screening, leaving 52 records for full-text retrieval. Of these, five could not be retrieved, one was considered a duplicate, and six were excluded based on the predetermined eligibility criteria, leaving 36 studies for inclusion, consisting of eight human studies and 28 animal studies.

### 3.1. Human Studies

#### 3.1.1. Biofunctional Molecules Involved in Enhancing OTM

The studies included in this systematic review assessed the efficacy of various biomolecules in accelerating orthodontic tooth movement (OTM) through local administration. The biofunctional molecules examined included Calcitriol—a vitamin D3 metabolite [17,18,19,20], Prostaglandin E1 [21,22], Vitamin C [23], and Recombinant human Relaxin [24].

The results were variable across substances and studies, as well as the study design, demographic details, sample size, study duration, intervention specifics (including the substance used and its administration pathway), applied force magnitude, tooth movement type, administration frequency, dosage, and outcome measures. The specific details can be found in Table 3 and Table 4.

##### Calcitriol

Calcitriol emerged as a prominent biofunctional molecule capable of improving the OTM rate in four original studies; in two studies [18,20] it produced statistically significant outcomes in enhancing OTM, while the remaining two showed acceleration of OTM, although not all reached statistical significance [17,19]. The action of Calcitriol is multifaceted, with a powerful influence on the process of bone metabolism, contributing to both bone resorption and deposition, significantly enhancing the rate of OTM compared to control groups. In more depth, the stage of osteoblast development determines how much Calcitriol affects bone turnover. Normal vitamin D levels have been shown to reduce the receptor activator of the nuclear factor kappa-B ligand/osteoprotegerin (RANKL/OPG) ratio and reduce osteoclastic bone resorption through vitamin D receptors in mature osteoblasts [9,25,26]. Likewise, Calcitriol acts on fully developed osteoblasts by enhancing the bone apposition rate. However, higher concentrations of Calcitriol stimulate osteoclastic bone resorption by increasing the RANKL/OPG ratio in less developed osteoblasts. The impact of Calcitriol is associated with the augmentation of RANKL expression by nearby cells, thus leading to the stimulation of osteoclasts [26,27,28].

In line with these biological pathways, Al-Hasani et al. [17] investigated the efficiency of Vitamin D3 (25 pg/0.2 mL) injections to accelerate maxillary canine retraction over 3–6 months in a 17-patient trial. The intervention demonstrated no additional root resorption or adverse alveolar bone effects, confirming its safety. Remarkably, it achieved a 51% acceleration in tooth movement on the experimental sides, illustrating a favorable periodontal response and validating a prior human trial [20], where 15 patients received weekly Vitamin D3 injections in varying dosages over six months. The study found a dose-dependent effectiveness, with a 25 pg dose leading to a 51% faster canine movement rate. The absence of detrimental effects on the PDL emphasizes the safety and efficiency of Calcitriol in orthodontic applications.

Varughese et al. [18] administered monthly local periodontal injections of calcitriol (50 pg/0.2 mL), noting a statistically significant increase in the amount of canine distalization over three months while Iosub Ciur et al. [19] investigated patients that received weekly Vitamin D3 injections over three months. The intervention showed no root resorption and resulted in 70% more tooth movement on the experimental side.

##### Prostaglandins

Prostaglandin E1 (PGE1) was consistently effective, with both studies reporting a statistically significant increase in OTM. This biofunctional molecule’s role in accelerating OTM is likely through its impact on bone remodeling, particularly by enhancing the bone resorption necessary for tooth movement [5]. PGE1 can act as an intermediary in transmitting mechanical stress during orthodontic tooth movement. It can enhance bone resorption, reduce collagen synthesis, and elevate cAMP (cyclic adenosine monophosphate) levels. Bone resorption is accentuated by promoting osteoclastogenesis and activating pre-existing osteoclasts. PGE1 also increased the levels of matrix metalloproteinase-8 (MMP-8), resulting in a reduction of procollagen, necessary for the remodeling of bone and the PDL [8,9,29].

The study conducted by Patil et al. [21] used PGE1 injections, revealing no adverse macroscopic or radiographic effects. It demonstrated a substantial enhancement in tooth displacement, with the intervention side moving 1.7 times faster than the control side. This significant acceleration highlights PGE1’s potential as a safe and effective orthodontic adjuvant, with results in line with Yamasaki’s et al. [22] PGE1 study, who observed that the rate of canine movement was almost 1.6-fold higher on the PGE1-injected side compared to the vehicle-injected side, with no significant side effects reported.

##### Vitamin C

Vitamin C, although studied in a single trial, presented a statistically significant outcome, enhancing the rate of impacted maxillary canine movement. Vitamin C’s role in collagen synthesis and its influence on bone and soft tissue healing may account for its efficacy in accelerating OTM [30]. Vitamin C affects osteoclastic activity, osteogenesis, tissue repair, and PDL organization. It boosts collagen type I synthesis, a key component of bone matrix and PDL, as well as bone mineralization, collagen type X production, alkaline phosphatase expression, and osteoblast development and differentiation, leading to bone regeneration [30,31].

The trial conducted by Yussif et al. [23] investigated the use of Vitamin C submucosal injections over 12 months in the traction and alignment of impacted canines. The intervention showed no evidence of bone or root resorption and led to clinically significant improvement in movement rate. These findings indicate Vitamin C as a potent catalyst for tooth eruption, maintaining PDL integrity.

##### Recombinant Human Relaxin

Conversely, Recombinant human Relaxin did not demonstrate a significant change in OTM under the tested conditions. Despite its known influence on collagen turnover and potential effects on the periodontal ligament, the dosages or methods of administration used in the study may not have been sufficient to alter tooth movement rates [24].

**Table 4:** Side effects and outcomes of the analyzed biofunctional molecules.

Authors, Year	Biofunctional Molecules	Description of Groups, Dosage	Side Effects	Outcome
1. AlHasani et al., 2021 [17]	Calcitriol	EG: OTM + 25 pg/0.2 mL Vitamin D3 diluted in DMSO CG: OTM + no intervention	The intervention did not result in additional root resorption and did not adversely affect the integrity of the alveolar bone (*p* > 0.05 in canine root resorption evaluations between the experimental and control sides).	The local injection of vitamin D3 improved canine retraction and led to a more favorable periodontal response. The study validated and referenced previous human studies demonstrating a 51% acceleration in tooth movement on the experimental sides.
2. Varughese et al., 2019 [18]	Calcitriol	EG: OTM + 50 pg per 0.2 mL of calcitriol (1,25 DHC) gel CG: OTM + 0.2 mL placebo gel	The findings indicated a statistically significant decline in cancellous bone density on the experimental side in comparison to the control side. There also was a significant overall decline in bone density of the cortical bones during OTM, on both experimental and control sides.	Quantitative measurement of canine distalization revealed a highly significant difference (*p* < 0.001) between the experimental and control sides over a period of three months. During the second and third months, there was a notable increase in the rate of canine movement on the experimental side, which was statistically significant.
3. Iosub Ciur et al., 2016 [19]	Calcitriol	EG: OTM + 42 pg/mL–0.2 mL of vitamin D3 diluted in DMSO CG: OTM + no intervention	There was no evidence of root resorption observed three months after the initial treatment with vitamin D3, as assessed by cone-beam CT examination.	The mean rate of tooth displacement was higher in the experimental side compared to the control side. The experimental teeth exhibited an average of 70% more pronounced tooth movement compared to the control teeth. The disparities between the two quadrants (control and experimental) exhibited statistical significance (*p* < 0.0313).
4. Al-Hasani et al., 2011 [20]	Calcitriol	EG1: OTM + 15 pg calcitriol diluted in 0.2 mL DMSO EG2: OTM + 25 pg calcitriol diluted in 0.2 mL DMSO EG3: OTM + 40 pg calcitriol diluted in 0.2 mL DMSO CG: OTM + placebo (0.2 mL DMSO)	The periapical radiographs revealed no detrimental impact of calcitriol on the periodontium.	The study found that, when calcitriol is injected locally, its effectiveness and cost efficiency in humans followed a dose-dependent pattern. Specifically, a dose of 25 pg of calcitriol resulted in a 51% faster rate of experimental canine movement compared to the control group. Additionally, doses of 15 pg and 40 pg each led to approximately a 10% acceleration in orthodontic tooth movement.
5. Patil et al., 2005 [21]	Prostaglandin E1	EG: OTM + 1 g PGE1/1 mL lignocaine CG: OTM + placebo (lignocaine only)	No adverse effects were noticed, neither macroscopically nor through X-ray imaging, in the area where PGE1 was injected during the experimental study and the two-year follow-up period of active orthodontic treatment.	The observed data clearly demonstrated a substantial enhancement in orthodontic tooth displacement on the side injected with PGE1, compared to the control side. The ratio of the intervention side to the control side was 1.7/1, as seen during a period of one month.
6. Yamasaki et al., 1984 [22]	Prostaglandin E1	EG: OTM + 10 µg PGE1 CG: OTM + placebo (lidocaine only)	No adverse effects were observed in the surrounding tissues when examined macroscopically and radiographically.	Local administration of 10 µg of PGE1, in the gingiva adjacent to the orthodontically treated teeth resulted in a significant increase in the rate of tooth movement compared to the control side. The distal canine movement rate was nearly 1.6 times higher on the side where PGE1 injections were administered compared to the side where the vehicle was injected. This difference was statistically significant (*p* < 0.05).
7. Yussif et al., 2018 [23]	Vitamin C	EG: OTM + vitamin C with a dosage calculated for a single tooth * CG: OTM + no intervention	There was no evidence of bone or root resorption in the teeth that were treated after receiving a vitamin C injection.	The intervention group showed a clinically significant improvement in movement rate (2–2.5 mm), while the control group had a lower rate (0.5–1.5 mm). The p-value was found to be *p* < 0.003. The results implied that locally administered vitamin C is a powerful catalyst for eruption that has the benefit of preserving the integrity of the periodontium.
8. McGorray et al., 2012 [24]	Recombinant human Relaxin	EG: OTM + 50 mg Relaxin divided in 2 injections of 0.1 mL CG: OTM + placebo vehicle	None of the subjects exhibited any presence of anti-Relaxin antibodies at any given period and no side effects were noted.	There were no statistically significant differences in tooth displacement between the experimental and the control group.

OTM—orthodontic tooth movement; DMSO—Dimethyl sulfoxide; PGE1—Prostaglandin E1. * Dosage calculated according to the author’s previous study [32].

#### 3.1.2. Risk of Bias Assessments

Risk of Bias Assessments across the studies varied, with several studies demonstrating moderate risk due to small sample sizes, lack of detailed randomization, blinding processes, and potential confounding factors not being thoroughly controlled. The studies that provided statistically significant results offered promising evidence towards the efficacy of pharmacological agents in accelerating OTM. These factors necessitate a cautious interpretation of the results and underscore the need for further research with larger sample sizes and more rigorous methodologies.

**Figure 2:**
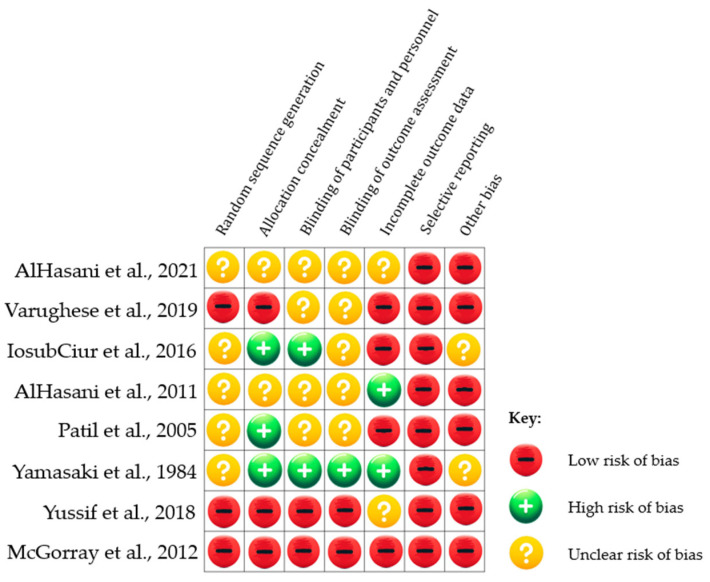
Risk of Bias assessment [17,18,19,20,21,22,23,24].

In more detail, the results of the RoB assessment can be found in Figure 2.

The collective findings of these human studies suggest that certain pharmacological agents, particularly those influencing bone metabolism, can enhance the rate of OTM. However, the studies’ limitations, particularly related to their design and execution, indicate that even though the evidence is promising, it is not definitive. Further investigation with more robust clinical trials is warranted to confirm these agents’ efficacy and safety in clinical practice.

### 3.2. Animal Studies

In vivo animal studies evaluated numerous substances that could accelerate OTM (n = 28), out of which 27 had a local (injection—submucosal or intraligamentary) administration route and only one was applied topically (transmucosal absorption).

Prostaglandin E1 and E2 [32,33,34,35,36,37], calcitriol [28,32,38], RANKL and RANKL expression plasmid [39,40,41], growth factors such as epidermal growth factor [42,43], recombinant human insulin-like growth factor-1 [44], basic fibroblast growth factor (bFGF) [45], macrophage colony-stimulating factor (M-CSF) [46], PTH [47,48], osteocalcin [49,50], vitamin C and E [51], biocompatible reduced graphene oxide [52], exogenous thyroxine [53], sclerostin protein [54], a specific EP4 agonist (ONO-AE1-329) [55], carrageenan (CGN) [56] and herbal extracts [57,58] were evaluated in the researched studies.

Species, sample, OTM and force, frequency of administration, and outcomes vary between studies, but most studies show statistically significant OTM acceleration in experimental groups compared to control groups. Table 5 contains a more detailed breakdown of the stated aspects.

#### 3.2.1. Prostaglandin E1 and E2

Prostaglandins alter bone metabolism and promote bone formation under mechanical stimulation by promoting osteoblast proliferation, both progenitor and mature. Applying mechanical strain to bone can increase bone resorption through increasing the osteoclastic population. Integrating orthodontic force with exogenous prostaglandin E2 has a combined effect on alveolar bone, enhancing both resorption and deposition [32,59].

Caǧlaroǧlu et al. [34] compared the effects of prostaglandin E2 (PGE2) on orthodontic tooth movement and bone metabolism in New Zealand rabbits (n = 45). PGE2 was administered via intravenous, submucosal, or intraligamentous routes after appliance insertion and on days 1, 3, 7, and 14 with a total dose of 1.2 μg. Results showed tooth movement in both the experimental and positive control groups, but the intraligamentous PGE2 group had the highest values of all analyzed parameters, including serum calcium and phosphorus levels and osteoclastic and osteoblastic populations, concluding that submucosal and intraligamentous PGE2 administration significantly increased orthodontic tooth movement and bone metabolism.

Kale et al. [32] investigated the effects of calcitriol (1,25 DHCC) administration on days 0, 3, and 6 and PGE2 once, immediately after establishing the rat OTM model (n = 37), with a 1,25 DHCC dosage of 20 μL of 10^−10^ mol/L and a PGE2 dosage of 0.1 μg/0.1 mL. Although tooth movement was relatively similar, the PGE2 group had much more osteoclastic activity than the 1,25-DHCC group. In contrast, the 1,25-DHCC group had a much higher number of osteoblasts on the alveolar surface. This research demonstrates that 1,25-DHCC promotes bone formation more effectively than PGE2, facilitating the relationship between formation and resorption in alveolar bone reconfiguration during OTM.

Seifi et al. [35] investigated the effects of local injections of prostaglandin E2 (PGE2) alone and with calcium gluconate (Ca) on orthodontic tooth movement (OTM) and root resorption. The rodents (n = 24), divided into experimental and control groups, received 0.1 mL of 1 mg/mL PGE2 submucosal injections, with or without an intraperitoneal injection of 200 mg/kg Ca (10%) in addition to PGE2. All injections were performed on days 0 and 7. The results showed a significant acceleration in OTM after PGE2 injection compared to the control group. Although Ca lowered OTM, a substantial increase (*p* < 0.05) was still seen. Root resorption differed significantly (*p* < 0.05) between the PGE2 and normal groups. The PGE2 group showed a considerable increase in root resorption compared to the normal group. There were no statistically significant changes in root resorption between the PGE2 + Ca and the normal or control groups. The findings demonstrate the role of calcium ions functioning in conjunction with PGE2 in regulating root resorption while considerably enhancing OTM.

Cui et al. [33] investigated the effect of injecting akebiasaponin D (ASD) at different doses locally on the speed of orthodontic tooth movement in rats (n = 40) compared to the effectiveness of PGE2 administration. Both substances were administered once, immediately after establishing the OTM model. The ASD solution was administered at varying concentrations, with a dosage of 5 mg/kg in the ASD1 group and 10 mg/kg in the ASD2 group. A solution of PGE2 was administered at a dosage of 25 μg/kg in the PGE2 group. The results indicated a progressive increase in the distance between the first and second molars, as compared to the control group. Nevertheless, there was no notable disparity observed between the ASD2 group and the PGE2 group. Osteoclast numbers exhibited an increase on the side experiencing tension, reaching their highest point on the 21st day, and thereafter declined. The study determined that the local administration of ASD solution can accelerate the process of orthodontic tooth movement. It was found that a dosage of 10 mg/kg of ASD solution is as effective as the PGE2 solution.

These studies confirm the findings of Leiker et al. [36] which evaluated the long-term effects of varying concentrations and frequencies of injectable, exogenous prostaglandin E2 (PGE2) on tooth movement and root resorption. In his study, 132 rats were divided into two experimental time periods of 2 and 4 weeks, and divided into four subgroups based on concentration levels of PGE2 injections. Half of the dosage subgroup received a single injection at appliance placement, while the other half received weekly injections. The results showed that injections of exogenous PGE2 over an extended period enhanced orthodontic tooth movement in rats. However, there was no statistically significant difference in tooth movement between the single and multiple injection groups or among the four concentration levels used in either the 2- or 4-week time periods. The amount of root resorption increased with the use of prostaglandin injections, specifically with increased numbers of injections and concentrations of PGE2. The study determined that a lower dosage of PGE 2 (0.1–1.0 µg) is enough to improve OTM. Similar results were also found by Yamasaki et al. [37], who demonstrated that combining local prostaglandin E1 or E2 with orthodontic tooth movement can nearly double the rate in monkeys compared to a control group. There were no macroscopical negative effects seen in the gingiva or related structures, after 40 µg PGE1 or PGE2 submucosal injections on days 0, 1, 5, 9, 12, and 15.

#### 3.2.2. Growth Factors

In the context of OTM, growth factors are involved in angiogenesis and therefore play an important role in increasing the rate of activation of osteoclasts. Peng et al. [44] explored the effects of Recombinant human insulin-like growth factor-1 (rhIGF-1) in bone remodeling and orthodontic tooth movement using submucosal injections every two days in a rodent model (n = 80). The results demonstrated that the rhIGF-1 group had more bone resorption and lacunae on the alveolar bone than the control group, with a considerably higher number of osteoclasts in the compression side of the periodontal ligament than the control group. The distance of tooth movement in the rhIGF-1 group increased significantly during the experiment, indicating that rhIGF-1 enhanced OTM.

Similarly, the effect of basic fibroblast growth factor (bFGF) was investigated by Seifi et al. [45], who found out that a submucosal injection of either 0.02 cc of 10 ng, 100 ng, or 1000 ng was effective in a dose-dependent manner, with rats (n = 50) receiving the most effective injection at 1000 ng. The current study found that local application of angiogenic factors such as bFGF can shorten the time of orthodontic therapy.

Alves et al. [42] and Saddi et al. [43] in their rodent model (n = 96, respectively, n = 32) both evaluated the influence of epidermal growth factors (EGF) injected submucosally once, immediately after establishing the mouse OTM model, in different concentrations, with the aim of accelerating orthodontic tooth movement. The findings confirm that EGF-liposome treatment can enhance tooth mobility and osteoclast numbers with statistically significant results compared to controls. This was associated with high RANKL expression, inducing osteoclast genesis and accelerating OTM [42].

On the other hand, Brooks et al. [46] determined that a 14% increase in tooth movement rate can be achieved in a mouse model (n = 48) with a single subperiosteally injection of macrophage colony-stimulating factor—an early osteoclast recruitment/differentiation factor (M-CSF), suggesting it may be a viable choice in therapeutic settings. M-CSF could be injected during appliance activation or reactivation, thus reducing treatment duration without increasing appointments with the orthodontist. Additionally, administering M-CSF may allow for lower force values, reducing the risk of undesired root resorption.

#### 3.2.3. RANKL and RANKL Expression Plasmid

RANKL and RANKL expression plasmids play a role in orthodontic tooth movement through their ability to enhance osteoclast activation and bone degradation by attracting cytokines. Chang et al. [39] investigated the effect of the receptor activator of nuclear factor kappa-B ligand (RANKL) formulated in microspheres (RANKL formulation with PLGA-aqueous hydroxyethyl cellulose microspheres—1.5 mg/4.5 μL), injected through flapless osteoperforations in a rat model (n = 24), on orthodontic tooth movement. The study discovered that injectable RANKL formulations were successful in accelerating bone resorption in comparison to other control groups. There was a notable increase in tooth movement (129.2% more tooth movement than without formulation and 71.8% more than with placebo formulation) and a statistically significant increase in TRAP activity (an increase of the amount of osteoclast activity determined by using tartrate-resistant acid phosphatase staining). The groups did not show any noticeable differences in root resorption. The study’s findings indicate that injectable RANKL formulations have a substantial positive impact on (OTM).

Similarly, Li et al. [40] used RANKL, injected daily subperiosteally for 2 weeks in a larger rodent model (n = 60) in order to assess its ability to enhance OTM. The study found that the experimental group exhibited a statistically significant increase in tooth movement and reduction in bone volume on days 14 and 21. Additionally, the number of osteoclasts increased on days 3, 7, 14, and 21 but there was no notable difference at the end of the experimental period. Significantly elevated levels of RANKL expression were detected on days 7 and 14, accompanied by notable alkaline phosphatase activity. No substantial systemic alterations were detected.

Kanzaki et al. [41] evaluated the efficacity of RANKL expression plasmid (5-μL) in a hemagglutinating virus of Japan (HVJ) envelope vector, injected subperiosteally in a rat tooth movement model (n = 25). Local transfer of the RANKL gene increased the expression of RANKL and the formation of osteoclasts in the periodontal tissue in a statistically significant manner, without causing any systemic side effects.

#### 3.2.4. Calcitriol

Calcitriol (a Vitamin D3 derivate), or 1,25 DHC, supports alveolar bone remodeling by enhancing the proliferation and function of the osteoblasts, with a higher rate of recruitment and stimulation of mononuclear osteoclasts, leading to enhanced bone resorption on the pressure side of the periodontal ligament [28]. Takano Yamamoto’s [38] study on Wistar Rats (n = 40), as well as the experiment conducted by Collins et al. [28] on 10 young adult cats determined its role in enhancing orthodontic tooth movement with an increase varying from 60% (weekly intraligamentary injection of 0.1 mL of DMSO containing 50 µg/mL of 1,25D) [28] up to 245% (submucosal injections every 3 days of 1,25-(OH)2D3 20 μL of 10⁻10 mol/L) [38], their findings being consolidated by the human studies previously mentioned [17,18,19,20].

#### 3.2.5. PTH

The parathyroid hormone, when applied directly to the areas of compression of the PDL, has the potential to enhance the activity of osteoblasts and indirectly affect osteoclasts. Lu et al. [47] attempted to determine the effects of local administration (injections into a micro-osteoperforation) of parathyroid hormone (PTH) or parathyroid hormone-related protein (PTHrP) carried by PECE (thermosensitive PEG-PCL-PEG) hydrogel on osteogenesis and osteoclastogenesis during orthodontic tooth movement on a rodent model (n = 40). At 14 days, the total release of PTH or PTHrP from PECE hydrogels exceeded 75% on a sustained basis. The distance of OTM in the PTH or PTHrP group statistically significant compared to the control groups. PTH injections were also able to lower the rate of orthodontic relapse concluding that local injection of either PTH or PTHrP delivered via controlled release enhances OTM by controlling periodontal bone remodeling.

Soma et al. [48] also evaluated the potential of a slow-release formula of PTH injected subperiosteally every other day through the rat (n = 56) experiment in order to accelerate OTM, with statistically significant results. The experiment determined that PTH dissolved in methyl cellulose gel (MC gel) injections at 1 μg/400 g body weight caused a 1.6-fold increase in the rate of tooth movement.

#### 3.2.6. Other Biosubstances

Osteocalcin [19,20], vitamin C and E [21], biocompatible reduced graphene oxide [22], exogenous thyroxine [23], sclerostin protein [24], a specific EP4 agonist (ONO-AE1-329) [25], carrageenan (CGN) [26], and herbal extracts [27,28] were also investigated.

The proteins examined demonstrated their capacity to recruit and differentiate osteoclasts, hence playing a role in bone remodeling. Lu et al. [54] evaluated the ability of sclerostin protein to accelerate orthodontic tooth movement in Wistar Rats (n = 48), showing that injecting sclerostin protein submucosally into the alveolar bone on the compression side enhances the formation of osteoclasts via stimulating osteoclastogenesis. The results demonstrated a significant increase in tooth movement in the groups that received sclerostin injections of 4 μg/kg and 20 μg/kg. Additionally, the group receiving a dosage of 20 μg/kg experienced a notable reduction in BV/TV (bone volume fraction) and an increase in the number of TRAP-positive osteoclasts at the site of compression. In animal studies (rodent models, n = 88 and n = 33) on the effects of calcitonin (a major noncollagenous bone matrix protein), local injections were conducted by Hashimoto et al. [49] and respectively by Kobayashi et al. [50], with both studies showing statistically significant results compared to the control groups both in amount and rate of tooth movement. Additionally, histological analysis demonstrated that the injections significantly enhanced the presence of osteoclasts on the compressed side of the alveolar bone surface. The findings indicate that osteocalcin has a supplementary impact on the speed of orthodontic tooth movement by promoting the formation of osteoclasts on the side experiencing pressure [50].

Jiao et al. [52] assessed the impact of biocompatible gelatin reduced graphene oxide (GOG) on the process of bone remodeling, osteoclastogenesis, and angiogenesis, as well as its ability to speed up orthodontic tooth movement. The mouse model experiment (n = 12) showed that local injections of GOG resulted in an increased rate of tooth movement, along with more bone resorption by osteoclasts and the formation of new blood vessels, as compared to control groups. Furthermore, when locally injected around teeth, the buildup of GOG in the spleen did not have any adverse effects on animal survival and lifespan. The results illustrate the effectiveness of GOG in promoting bone remodeling and OTM while ensuring biosecurity and biological functionality.

In a 4-week dog OTM model (n = 8), Jung et al. [53] tested a transmucosal administration (via tablets bonded on the orthodontic appliance and replaced once a week) of exogenous thyroxine, and found that although the experiment did not have a statistically significant effect on the rate of OTM, microscopic evaluation at the end of the experimental period revealed an increase in the osteoclasts number.

Chung et al. [55] aimed to examine the combined enhancement of tooth mobility and bone growth in vivo by stimulating the prostaglandin receptor EP4. The rat experiment (n = 25) investigated the local administration of a particular EP4 agonist (ONO-AE1-329) via injection to replicate the effects of ligand binding. To assess tooth movement and bone volume, researchers employed soft X-ray and micro-computed tomography. The results indicated that the administration of the EP4 agonist alone did not have a significant impact on body weight, macro-structures, or bone volume when compared to the group treated with only vehicle or control side. Nevertheless, the administration of the injection, when combined with tooth movement, enhanced both tooth movement and the volume of bone in the alveolar tension side, obtaining statistically significant results.

Lastly, Carrageenan (CGN), a seaweed-derived common polysaccharide food additive and two different herbal extracts (Asperosaponin VI and dried root aqueous extract of Salvia miltiorrhiza) were evaluated in order to assess their ability to influence OTM.

Kavoli et al. [56], in their rat model experiment (n = 28), revealed that CGN has the ability to trigger inflammation (through increased osteoclastic activity), which could potentially accelerate OTM. The findings indicate that carrageenan injected locally can induce inflammation and increase the rate of OTM, which may enhance the efficacy of orthodontic treatment, with a statistically significant difference between the experimental and the control groups. The authors state that carrageenan shows promise as a viable aid in OTM, nevertheless mentioning that additional research is necessary to comprehensively grasp its potential advantages. Similarly, Xiao et al. [58] had statistically significant results when evaluating the potential of a Chinese herbal aqueous root extract (Salvia miltiorrhiza—ESM) as well as of its main component—Danshensu. The rat experimental model (n = 150) also observed an increase in the levels of RANKL and OPG in the treatment groups as compared to the control group. Nevertheless, OPG expression increased at a slower rate compared to that of RANKL. The levels of RANKL dramatically dropped when no orthodontic pressure was applied, particularly in the ESM groups, which indicates that ESM might accelerate periodontal changes by osteoclasts proliferation. Another natural biomolecule, Asperosaponin VI (ASA VI), was evaluated by Ma et al. [57] in order to assess its effects on OTM. The study conducted on a rat model (n = 64) found that ASA VI enhanced tooth movement in the experimental group, increased the count of osteoclasts, and improved the expression of osteoclast differentiation factors in comparison to the control group. The research team’s conclusion states that ASA VI local administration could accelerate tooth movement by enhancing the activity of osteoclasts, which in turn promotes bone resorption on the compression side, while at the same time exerting a beneficial impact on bone formation on the tension side.

#### 3.2.7. Risk of Bias Assessment

**Figure 3:**
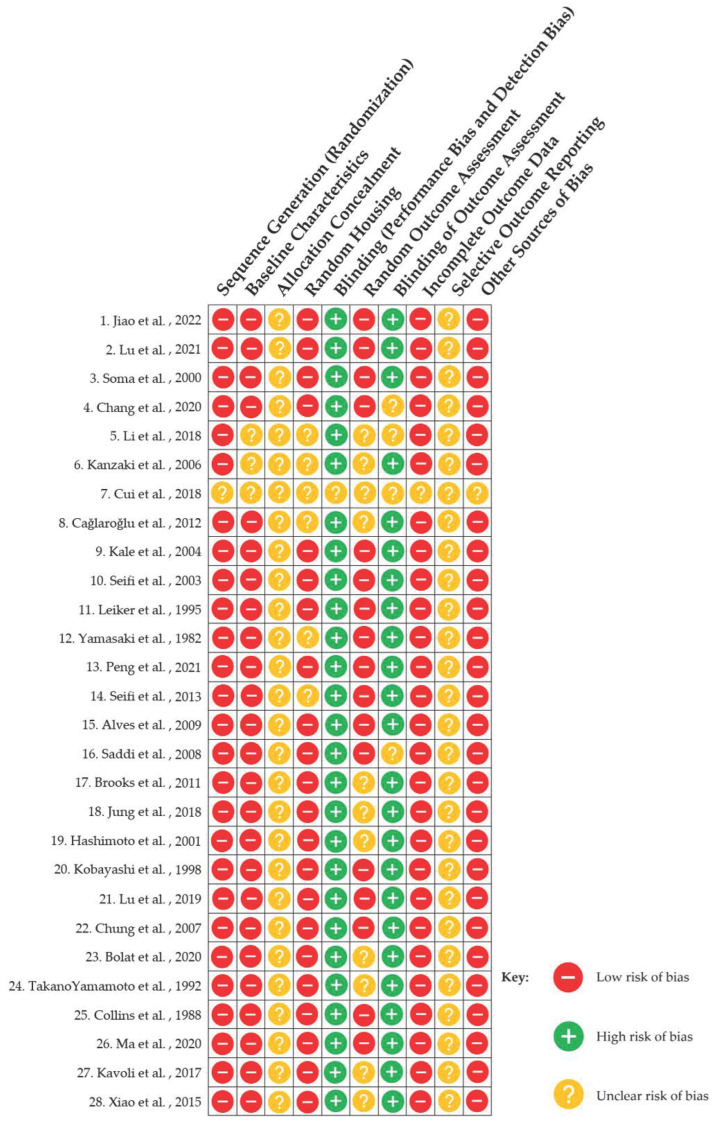
Risk of Bias assessment [28,32,33,34,35,36,37,38,39,40,41,42,43,44,45,46,47,48,49,50,51,52,53,54,55,56,57,58].

The SYRCLE’s risk of bias assessment (Figure 3) [60] for the included 28 animal studies revealed that while the studies differed in certain methodological areas, the majority had comparable risk profiles across specific bias categories.

The majority of the animal studies had a low risk of randomization bias, indicating that the separation of animals into treatment and control groups was often performed correctly. Most studies verified that the animals used had comparable baseline characteristics, resulting in a minimal risk of bias in this area. Allocation concealment was a consistent source of confusion throughout the trials, posing a medium to high risk of bias. The majority of the studies were classified as low risk in random housing, implying stable and regulated living conditions among experimental groups. Most studies did not provide appropriate information on whether treatment delivery and outcome assessment were blinded, raising concerns about data interpretation. The techniques of outcome measurement were typically consistent and methodical across trials, resulting in a low risk of bias in this area. Blinding during therapy administration and a lack of blinding in outcome assessment were common, indicating a significant risk of bias that might impair the reliability of the findings. Most studies provided detailed data on animal follow-up and tracked all animals throughout the experimental period, indicating a minimal likelihood of attrition bias.

Despite the need for more robust and standardized study designs, particularly in terms of improving blinding procedures and increasing transparency in allocation concealment and outcome reporting, the majority of the studies evaluated show a low risk of bias in critical areas such as randomization, baseline characteristic uniformity, and outcome data completeness. This conclusion confirms the dependability of the animal study findings in this sample, while the highlighted areas for improvement indicate that more severe methodological requirements are required to further strengthen the scientific rigor of future research in this subject.

## 4. Discussion and Perspectives for Enhancing OTM

### 4.1. Biomolecules Involved in OTM

In order to improve OTM, local biomolecule delivery has been extensively studied [3,4,14]. The results of this systematic review highlight an area where significant differences in biofunctional molecules’ efficacy were found. Notably, no studies have examined the topical administration of these biosubstances in order to enhance the OTM, a gap that contrasts with the recent advances in the periodontal regeneration field, where the use of hydrogels has emerged as a possible local delivery method that might be useful in maintaining the stability of the therapeutic drug levels [61,62]. The absence of such studies in the OTM acceleration field may be attributable to the lack of suitable formulations that facilitate topical application while ensuring the bioavailability and stability of the biofunctional molecules and substances.

The implications of these findings are twofold. Firstly, the authors affirmed the potential of certain biofunctional molecules evaluated during human experimental studies, such as Calcitriol and PGE1, to significantly enhance tooth movement, consistent with their roles in bone metabolism. Vitamin C also emerged as a promising candidate, particularly in the acceleration of impacted maxillary canine movement. However, the efficacy of Recombinant human Relaxin remains questionable under the tested conditions, potentially due to inadequate dosing or methodological constraints.

Among the previously mentioned biofunctional molecules, 1,25 DHC, PGE1, and vitamin C were examined in both human and animal models to determine their effectiveness in improving OTM. These substances have demonstrated encouraging outcomes, with each possessing its own set of advantages and disadvantages. Animal studies offer several benefits, including a controlled environment, ethical considerations, standardization, and detailed histological analysis. They provide an initial understanding of potential consequences, but there are species differences, translation to humans, ethical concerns and a limited adaptability. Human studies, on the other hand, directly answer clinical questions, account for individual variation, provide the possibility for a long-term follow-up, and include patient-reported outcomes. They also follow strict ethical guidelines, ensuring informed consent while minimizing harm. However, they face challenges such as ethical constraints, sample size issues, variability due to genetics, lifestyle, compliance, and other factors.

The translation of research findings into clinical practice requires bridging the gap between these animal experimental models and possible human studies. This involves addressing the physiological differences, OTM mechanisms, and treatment responses observed in animals and humans. It also encourages careful consideration of the ethical implications of using certain substances in humans based on the findings of animal studies.

Secondly, a number of molecules have shown dose-dependent results in animal models, indicating their potential for accelerating OTM. However, these substances, such as RANKL and RANKL expression plasmid, growth factors, PTH, osteocalcin, vitamin E, bio-compatible reduced graphene oxide, exogenous thyroxine, sclerostin protein, a specific EP4 agonist (ONO-AE1-329), carrageenan, and herbal extracts, have not yet been tested in human subjects. It is critical to discuss these molecules’ potential, mechanisms of action, and the consequences of their dose-dependent effects.

The substances that performed best in animal models—RANKL and RANKL expression plasmid, growth factors, PTH, and the reduced graphene oxide—showed a statistically significant enhancement of the OTM rate without systemic effects. The local effects of these substances and their distribution are essential for their potential use in orthodontics, as they enable targeted treatment with minimal side effects.

However, several gaps and limitations were identified in the existing studies that precluded the conduct of a meta-analysis. These include the use of different animal species (rats, mice, rabbits, cats, dogs, and monkeys), different sample sizes (n = 2 to n = 150), different sexes (male, female), a wide range of substances with an even wider range of dosages and a variety of administration routes (intraligamentary, submucosally or subperiosteally injections, as well as local injections into micro-osteoperforations and a transmucosal administration) and frequencies (varying from once—at the moment of establishing the OTM model, up to a daily administration for the entire experimental procedure).

The follow-up periods and outcome measurements also varied among the studies, with only a few including qualitative determinations such as histology analysis in addition to quantitative data. These differences in study design and methodology have resulted in varying evaluations of results, which, while posing a challenge for finding comparison and synthesis, also open up new pathways for future research.

### 4.2. Pharmacokinetics and Experimental Properties of the Investigated Pharmacological Substances

To fully assess the significance of the findings on the “off-label” use of biosubstances to enhance orthodontic tooth movement, it is necessary to examine the main indications for use, pharmacokinetics, and properties of the biomolecules investigated so far. This evaluation includes a thorough discussion of their clinical applications, biochemical properties, and physiological effects. The mechanism of action, dosages, administration routes, and outcomes of these biomolecules in terms of improving orthodontic tooth movement through local administration were thoroughly discussed in the results section. Here, we concentrate on their established pharmacological knowledge and application.

The scientific literature lacks detailed information on biomolecules that have not yet been used as conventional pharmacological substances (e.g., various growth factors such as bFGF, EGF, M-CSF, RANKL and its expression plasmid, osteocalcin, biocompatible reduced graphene oxide, sclerostin protein, ONO-AE1-329, CGN, and the aforementioned herbal extracts). This absence reveals a gap in our understanding and emphasizes the need for additional research.

Table 6 contains specific details about substances already used for pharmacological purposes, such as Prostaglandins E1 and E2, Calcitriol, PTH, exogenous thyroxine, and Vitamin C, such as their chemical formula, molecular type and weight, alternative names, indications and background, mechanism of action, absorption, metabolism, volume of distribution, protein binding, route of elimination, half-life, clearance, and toxicity. Furthermore, their experimental properties have been compiled, which include information on the biomolecules’ physical state, melting point (expressed in degrees Celsius), water solubility, and logP. All of this information was obtained from an electronic drug database [63]. A thorough search of the scientific literature for the previously mentioned biomolecules, which have not yet been used in conventional pharmacology, yielded no relevant results.

### 4.3. Future Research Perspectives and Directions

The findings indicate the need for future research to pivot towards the development of topical formulations for human application. Such formulations could revolutionize the patient’s experience by eliminating the discomfort associated with injections, reducing treatment costs, and minimizing the necessity for frequent clinical visits. Moreover, a topical approach could provide a more consistent and controlled release of the agent, potentially leading to improved treatment outcomes.

To effectively enhance the rate of OTM, the characteristics of an optimal biofunctional molecule must be meticulously defined. An ideal biofunctional molecule aimed at augmenting OTM should be delivered via a vehicle designed to ensure a controlled, slow release of the biomolecules. This enables sustained therapeutic action while minimizing the need for frequent reapplications. Biocompatibility and biodisponibility are paramount; the molecule must synergistically integrate with the biological system without eliciting any adverse general systemic or local, or tissue or cellular side effects.

Furthermore, it is crucial that the chosen biofunctional molecule does not disrupt cellular metabolism but rather selectively enhances the metabolic processes necessary for accelerating OTM. This selective augmentation should promote the delicate balance between bone apposition and resorption, which is crucial for effective and safe tooth movement. The molecule must be non-cariogenic and should maintain the physiological pH levels of saliva and mucosal tissues, ensuring no alteration to the oral microenvironment. Additionally, it should be devoid of oncological risks, reinforcing its safety profile.

From an applicability standpoint, the ideal biofunctional molecule should be administrable in a non-invasive manner, preferably allowing for self-application by the patient at home. This approach enhances patient compliance and convenience while reducing clinical visits. The formulation should be such that doses can be precisely calculated and tailored to individual patient needs, potentially monitored through measurable biomarkers in saliva or crevicular fluid. One such biomarker could be the crevicular MMP-8 levels, whose elevation in response to the biofunctional molecule could serve as a reliable indicator of therapeutic efficacy and dosage adequacy, since higher levels result in a reduction of procollagen, necessary for the remodeling of bone and the PDL.

Incorporating these characteristics into the future design and evaluation of biofunctional molecules will significantly advance the field. As this review underscores the necessity for robust, well-designed studies to explore such potential, the delineation of an optimal biofunctional molecule provides a structured pathway for future research. This approach not only promises to enhance the efficacy and safety of OTM but also aligns with the broader goals of patient-centered, minimally invasive orthodontic care.

While the examined studies offered a foundation for the therapeutic use of biofunctional molecules in orthodontic practice, the moderate risk of bias identified cautions against unequivocal endorsements. Small sample sizes, lack of detailed randomization, and blinding procedures underscore the necessity for more stringent research designs.

## 5. Conclusions

In conclusion, the evidence points to promising opportunities for enhancing OTM with biomolecules. The field is on the verge of translational research, with the development of effective topical formulations for human use being a critical area for future research, as well as advancements that could revolutionize orthodontic treatment by focusing on patient comfort and treatment effectiveness. Calcitriol, PGE1, and Vitamin C are, among others, biofunctional molecules capable of enhancing OTM, with promising results in both human and animal studies. The heterogeneity of study designs, species, sample sizes, and administration methods complicates result synthesis; however, these differences open up new pathways for research that could lead to standardized and robust study designs. A significant gap was also identified in the study of topical administration methods for these molecules, which may indicate an opportunity for future research to develop appropriate formulations that ensure bioavailability and stability for OTM enhancement.

## Figures and Tables

**Table 1:** PICOS and inclusion/exclusion criteria.

Domain	Inclusion Criteria	Exclusion Criteria
Population	Healthy human participants, undergoing any type of active OTM Animal studies, with healthy subjects undergoing any type of active OTM	Studies where participants suffered from systemic or syndromic conditions, or where these conditions were induced
Intervention	Local or topical administration of pharmacological agents capable to accelerate the rate of OTM	Studies where the aim was to inhibit OTM or enhance post orthodontic retention.
		Studies where, in order to manufacture the substance that could accelerate OTM, other invasive medical procedures, such as phlebotomy, were required (e.g., Platelet-rich plasma)
		The use of biomaterials and biosubstances that targeted other aspects of the orthodontic treatment such as reducing the associated pain
Comparison	Placebo intervention or no intervention	The use of a different substance or a different type of intervention for accelerating OTM (e.g., surgical interventions, low-level energy laser, vibration)
	Different dosages of the studied substance or different substances as long as there was a control group	
Outcome	Quantitative data regarding the amount of tooth movement measured by various methods	
	If possible, data also regarding the status of the root and surrounding tissues (PDL, alveolar bone)	
Study design	Experimental prospective controlled studies (randomized and non-randomized)	Non-comparative studies
	Clinical Trials	In vitro only or ex vivo studies
		Case reports, reviews, systematic reviews, meta-analyses, case series, opinion articles, letters from editors

OTM—orthodontic tooth movement; PDL—periodontal ligament.

**Table 2:** Research Keywords and MeSH terms.

Database/Register	Keywords and MeSH Terms
MEDLINE	(“tooth movement” [Title/Abstract] OR orthodontic* [Title/Abstract]) AND (pharmacological [Title/Abstract] OR drug* [Title/Abstract] OR prostaglandin* [Title/Abstract] OR vitamin [Title/Abstract] OR hormone [Title/Abstract] OR calcitriol [Title/Abstract]) AND (administration [Title/Abstract] OR local* [Title/Abstract] OR topical* [Title/Abstract]) NOT pain [Title/Abstract])
ProQUEST	Abstract (((“tooth movement” OR orthodontics OR “orthodontic treatment”) AND (pharmacological OR drug OR prostaglandin OR vitamin OR hormone OR substance) AND (administration OR use OR local OR topical) NOT pain)) OR summary((“tooth movement” OR orthodontics OR “orthodontic treatment”) AND (pharmacological OR drug OR prostaglandin OR vitamin OR hormone OR substance) AND (administration OR use OR local OR topical) NOT pain)))
Web of Science core collection	((“tooth movement” OR orthodontic OR orthodontics) AND (pharmacological OR drug OR drugs OR substance OR prostaglandin OR prostaglandins OR vitamin OR hormone OR calcitriol) AND (administration OR local OR locally OR topical OR topically) NOT pain))
Embase	(orthodontics:ti,ab,kw OR ‘tooth movement’: ti,ab,kw OR otm:ti,ab,kw) AND (prostaglandin:ti,ab,kw OR prostaglandins:ti,ab,kw OR vitamin:ti,ab,kw OR ‘vitamin d’:ti,ab,kw OR ‘vitamin e’:ti,ab,kw OR relaxin:ti,ab,kw OR hormone:ti,ab,kw OR calcitonin:ti,ab,kw OR calcitriol:ti,ab,kw OR pharmacological:ti,ab,kw OR drug:ti,ab,kw OR drugs:ti,ab,kw) AND (administration:ti,ab,kw OR local:ti,ab,kw OR topical:ti,ab,kw OR injection:ti,ab,kw)
Science direct	(((tooth AND movement) OR orthodontic) AND (pharmacological OR drug) AND (administration OR local OR topical)))
	(((tooth AND movement) OR orthodontic) AND (prostaglandin OR prostaglandins) AND (administration OR local OR topical))
	(((tooth AND movement) OR orthodontic) AND (hormone OR vitamin OR biological) AND (administration OR local OR topical)))
	((tooth AND movement) AND orthodontics) AND (administration OR local OR topical))
Scopus	TITLE-ABS ((“tooth movement” OR orthodont*) AND (pharmacol* OR drug* OR substance OR prostaglandin* OR vitamin OR hormone OR calcitriol) AND (administr* OR local* OR topical*) AND NOT pain)
	TITLE-ABS ((“tooth movement” OR orthodontic OR orthodontics) AND (pharmacological OR drug OR drugs OR substance OR prostaglandin OR prostaglandins OR vitamin OR hormone OR calcitriol) AND (administration OR local OR locally OR topical OR topically) AND NOT pain)
Cochrane CENTRAL	tooth movement orthodontic local administration NOT pain
Clinicaltrials.gov	condition or disease: ”tooth movement” other terms: orthodontic OR orthodontics

**Table 3:** Systematized data from retrieved studies.

Authors, Year, Study Design	Population, Age, (Sample)	Study Duration	Biofunctional Molecules	Administration Path	Applied Force (g), Movement	Frequency of Administration
1. Al-Hasani et al., 2021 [17], S-M	Patients who required bilateral maxillary 1st premolars extraction, 18 to 35 years of age, (17 patients—17 dental arches)	3 to 6 months	Vitamin D3—1,25 dihydroxycholecalciferol (1,25 DHC) (calcitriol—ampoules (Calcitriol, Mibe, Germany) diluted in DMSO	Injected locally into the distal periodontal sulcus of the canine	200 g, maxillary canine retraction (distalization)	Administration on each orthodontic visit (every three weeks) until extraction space closure, starting right before force application
2. Varughese et al., 2019 [18], S-M	Patients indicated for bilateral maxillary first premolar extraction, and minimum 5 mm of extraction space, 15 to 30 years of age, (15 patients—15 dental arches)	3 months	Vitamin D—1,25 dihydroxycholecalciferol (1,25 DHC) (calcitriol)	Local periodontal injection given distal to maxillary canine	150 g per side, maxillary canine retraction (distalization) with TPA as anchorage reinforcement	One monthly administration, starting right before force application
3. Iosub Ciur et al., 2016 [19], S-M	Patients who required bilateral extractions of the first premolars and need of bilateral distalization of canines, 13 to 34 years of age, (4 patients—6 dental arches)	3 weeks administration, 3-month study	Vitamin D3 (calcitriol—Decostriol^®^, Mibe Jena, Germany). Diluted in DMSO	Intraligamentary injection	150 g per side, canine retraction (distalization)	Administration once a week, for 3 weeks
4. Al-Hasani et al., 2011 [20], S-M	Patients who required bilateral maxillary 1st premolars extraction and bilateral maxillary canines retraction, 17 to 28 years of age, (15 patients—15 dental arches)	3 weeks administration, 5 weeks measurement, 6 months study	Vitamin D3 (calcitriol—(Calcitriol, Mibe, Germany diluted in DMSO)	Injected into the PDL on the distal side of canines	150 g per side, maxillary canine retraction (distalization) with TPA, ligation and stoppers as anchorage reinforcement	Administration once a week, repeated three times for every subject (at 1st, 2nd and 3rd visit)
5. Patil et al., 2005 [21], S-M	Patients that had undergone first maxillary premolar extraction, 13 to 25 years of age, (14 patients—14 dental arches)	60 days (2 months)	Prostaglandin E1 (PGE1)	Injection by local infiltration in the vestibular area at the upper canine region	150 per side, maxillary canines retraction (distalization) with TPA, molar stops and in 2 cases HG as anchorage reinforcement	Administration on the 1st day, 6th day and 17th day of the start of individual canine retraction
6. Yamasaki et al., 1984 [22], S-M	Patients who required first premolar extraction, 10 to 26 years of age, (25 patients—29 dental arches)	Phase 1: up to 33 days Phase 2: up to 21 days Phase 3: up to 4.8 months	Prostaglandin E1 (PGE1—CD, One Pharmaceutical Company, Ltd., Osaka, Japan)	Injections in the submucosal area of the buccal side of the right first premolar, maxillary and/or mandibular canines	Phase 1: 100 g per side, buccal movement of first maxillary premolars Phase 2 and 3: 150 per side, maxillary and/or mandibular canine retraction (distalization) with different methods used as anchorage reinforcement	Phase 1 and 2—administration at various intervals, starting from day 0, with a total injection number between 3 and 5 Phase 3—administration starting at day 0 and proceeding at approximately 10 days intervals, until space closure (1.5–4.8 months)
7. Yussif et al., 2018 [23], EG + CG	Patients with unilateral palatally positioned permanent canines, 15 to 40 years of age (12 patients)	12 months	Vitamin C	Submucosal injection (parallel to the mucosal surface within papillary connective tissue)	Impacted canine traction by elastic power chain activated every 2 weeks and alignment of said canine after its appearance in the buccal cavity	Administration repeated every 2 weeks until canine alignment
8. McGorray et al., 2012 [24], EG + CG	Patients needing minor incisor alignment of at least the maxillary incisors, 18 to 40 years of age, (39 patients)	8 weeks of tooth movement + 4 weeks of retention (total 3 months)	Recombinant human Relaxin	Injection to the gingival tissue to a depth of approximately 2 mm (lingual and buccal side)	Anteroposterior movement of 2 mm via a series of 4 maxillary aligners with 0.5 mm programmed of anteroposterior movement for the selected central incisor (only crown tipping)	Once, immediately after establishing the OTM model via 2 injections

S-M—Split Mouth Trial; EG + CG—Experimental group vs. Control group; DMSO—Dimethyl sulfoxide; TPA—Transpalatal arch; HG—Headgear.

**Table 5:** Animal studies included.

Authors, Year, Study Design	Species, Age, (Sample), Study Duration	Substance w/o Trade Name, Administration Path	Applied Force, Movement	Frequency of Administration, Description of Groups, Dosage	Outcomes—Amount of OTM for EG Compared to CG, %, *p*-Value
1. Jiao et al. [52], 2022, EG + CG	Mi (mice), 8-week-old male, (12), 10 days	Biocompatible reduced graphene oxide (biomaterial), buccal submucous local injection around the {…} molar	20 g, left first upper molar mesialization to incisor	Once, immediately after establishing the OTM model EG: OTM + 20 μL 10 mg/mL GOG solution CG: OTM + PBS	**amount of OTM**—n.a. (values presented in the article as an image) **%**—n.a. ***p* < 0.05**
2. Lu et al. [47], 2021, EG + CG	WR (rats), 7-week-old male, (40), 14 days	Parathyroid hormone (PTH) and parathyroid hormone-related protein (PTHrP), injected into a micro-perforation (MOP) with a diameter of 0.2 mm on the alveolar bone approximately 3 mm mesial to the maxillary first molar and 2 mm below the gingival margin	40 g, mesialization of upper first molars to incisors	Once, immediately applied after establishing the OTM model EG: OTM + PTH/PTHrP (R&D Systems) with the concentration of 1.25 μg/kg carried by PECE hydrogel CG: OTM + PBS/no intervention (gA: PTH/PBS gB: PTHrP/PBS gC: PTH/no intervention gD: PTHrP/no intervention gE(relapse group): PTH/PBS)	**amount of OTM**— PTH mean value 0.78 ± SD 0.06 mm PTHrP mean value 0.81 ± SD 0.04 mm no MOP group (mean value 0.46 ± SD 0.05 mm) MOP group (mean value 0.51 ± SD 0.04 mm) **%**—n.a ***p* < 0.005**
3. Soma et al. [48], 2000, EG + CG	WR (rats), male, (56), 12 days	Parathyroid hormone (PTH), local injection of PTH-MC (slow release formula) into the subperiosteum in the mesio-palatal region of the maxillary first molar	30 g, mesialization of the right maxillary first molar to incisors	The first injection was made immediately after wire placement, and carried on every other day CG1: OTM + no intervention EG2: OTM + vehicle dissolved in MC gel EG3: OTM + 0.1 µg PTH dissolved in MC gel EG4: OTM + 1 µg PTH dissolved in MC gel EG5: OTM + 1 µg PTH dissolved in 0.9% saline EG6: OTM + systemic injection EG7: no OTM + 1 µg PTH dissolved in MC gel	**amount of OTM**— PTH-MC injection at 1 μg/400 g body weight caused a 1.6-fold increase in the rate of tooth movement with a mean value 0.54 +/− SD 0.08 mm in the control group **%**—1.6-fold increase ***p* < 0.05** compared with vehicle in MC, ***p* < 0.01** compared with PTH in saline.
4. Chang et al. [39], 2020, EG + CG	WR (rats), 15-week-old male, (24), 14 days	Receptor activator of nuclear factor kappa-B ligand (RANKL) formulated in microspheres (RANKL formulation with PLGA-aqueous hydroxyethyl cellulose microspheres—1.5 mg/4.5 μL), injected palatal to the left maxillary first molar (flapless osteoperforation with a ¼ round bur and high-speed handpiece on the mesiopalatal of the left first maxillary molar; then sealed the gingiva with cyanoacrylate tissue adhesive)	5–8 g, left first upper molar mesialization to incisors	Once, immediately after establishing the OTM model EG: OTM + 1 μg RANKL—1 mg microsphere in 3 μL 10 per cent HEC gel, PCG: OTM + placebo microspheres (1 mg microsphere in 3 μL 10 per cent HEC gel) CG: OTM + no intervention	**amount of OTM**— RANKL formulation mean value 0.55 ± SD 0.25 mm Placebo formulation mean value 0.32 ± SD 0.1 mm No formulation mean value 0.24 ± SD 0.05 mm **%**—129.2% more tooth movement than no formulation and 71.8% more than placebo formulation ***p* < 0.05**
5. Li et al. [40], 2018, EG + CG	Mi (mice), 6-week-old male, (60), 42 days	Receptor activator of nuclear factor kappa B ligand (RANKL), injections subperiosteally administered to the buccal premaxillary bone […] under anesthesia	35 g, reciprocal force: buccal movement of upper incisors	Daily injections for 14 days EG: OTM + 0.04-μg/g (body weight) RANKL (Peprotech, Rocky Hill, NJ, USA) dissolved in 10-μL phosphate-buffered saline (PBS) CG: OTM + PBS	**amount of OTM**— control (0.52 ± 0.06 mm) and experimental (0.49 ± 0.07 mm) groups on day 7 (no significant difference) control group 0.79 ± 0.12 mm, experimental group 1.15 ± 0.27 mm on day 14 control group 1.07 ± 0.12 mm, experimental group 1.55 ± 0.22 mm on day 21 **%**—n.a. ***p* > 0.05** on day 7 ***p* < 0.05** on day 14 ***p* < 0.01** on day 21
6. Kanzaki et al. [41], 2006, EG + CG/S-M	WR (rats), 6-week-old male, (25), 21 days	RANKL expression plasmid, injected into the subperiosteal area, adjacent to the upper right first molar	g n.a., reciprocal force: palatal movement of first upper molars	Every 3 days until day 17 EG: HVJ envelope vector containing pcDNA-mRANKL 5-μL on the right side and PBS 5-μL on the contralateral side MG: OTM + mock vector solution 5-μL CG: no OTM + no intervention	**amount of OTM**— RANKL transfection side was 0.63 +/− 0.06 mm at 21 days control side 0.48 +/− 0.05 mm at 21 days **%**—per cent acceleration of TM (the RANKL transfection side/the control side) was 173.1, 137.8, 135.8 and 131.6% at days 3, 7, 14 and 21, respectively ***p* < 0.05** on day 3 and at the end of the experiment
7. Cui et al. [33], 2018 EG + CG	WR (rats), 6-week-old female, (40), 28 days	Akebiasaponin D (Chinese herbal extract) and PGE 2, local injection	40 g, mesialization of first upper molars	Once, immediately after establishing the OTM model EG1: OTM + 5 mg/kg ASD EG2: OTM + 10 mg/kg ASD EG3: OTM + 25 µg/kg PGE2 CG: OTM + PBS	**amount of OTM**—n.a. (values presented in the article as an image) **%** n.a. ***p* < 0.05** PGE2 on day 3 ***p* < 0.05** 10 mg/kg ASD group and 25 µg/kg PGE2 group on day 7 ***p* < 0.05** in all three groups compared to the control group on days 14, 21, 28 However, there was no significant difference (*p* > 0.05) between 10 mg/kg ASD group and 25 µg/kg PGE2 group
8. Caǧlaroǧlu et al. [34], 2012, EG + CG	NZR (rabbits), adult male, (45), 21 days	Prostaglandin E2 (PGE2), [PGE2 powder (p5640) was obtained from Sigma-Aldrich (St. Louis, MO, USA) and prepared as a 1 mg/mL stock solution by dissolving in ethanol] submucosal—immediately distal to the maxillary incisors, or intra ligamentous route—PDL surrounding the maxillary incisors, by using an insulin syringe or intraligamentous injector	20 g, reciprocal force: buccal movement of upper incisors	On days 0, 1, 3, 7, and 14, thereafter (total dose = 1.2 μg) EG1(gIII): OTM + PGE2 (10 μg/mL) 0.06-mL intravenous EG2(gIV): OTM + PGE2 (10 μg/mL) 0.06-mL submucosal EG3(gV): OTM + PGE2 (10 μg/mL) 0.06-mL intraligamenter CG1(NC-gI): no OTM + no intervention CG2(PC-gII): OTM + no intervention	**amount of OTM** EG1(gIII) a mean value 2.87 +/− SD 0.18 mm (min. 2.52—max 3.11 mm) EG2(gIV) a mean value 3.08 +/− SD 0.22 mm (min. 2.74—max 3.40 mm) EG3(gV) a mean value 4.54 +/− SD 0.31 mm (min 3.98—max 4.84 mm) CG2(PC-gII) a mean value 2.87 +/− SD 0.18 mm (min 2.52—max 3.11 mm) **%** n.a. EG1(gIII) ***p* > 0.05** EG2(gIV) ***p* < 0.001** EG3(gV) ***p* < 0.001**
9. Kale et al. [32], 2004, different EG + CG	SPR (rats), 6-week-old male, (37), 9 days	Prostaglandin E2 (PGE2, Sigma-Aldrich, St. Louis, MO, USA) and calcitriol—1,25-dihydroxycholecalciferol (1,25-DHCC, Roche, Basel, Switzerland), injected to the gingiva distal to the upper incisors	20 g, reciprocal force: buccal movement of upper incisors	1,25 DHCC on days 0, 3 and 6 and PGE2 once, immediately after establishing the OTM model EG1(gIV): OTM + 1,25 DHCC (20 μL of 10^−10^ mol/L EG2(gV): OTM + PGE2 0.1 μg —0.1 mL CG1(gI): no OTM + no intervention CG2(gII): OTM + no intervention CG3(gIII): OTM + dimethyl sulfoxide [DMSO] 20-μL (the vehicle of 1,25-DHCC)	**amount of OTM** CG2(gII) mean 1.72 +/− SD 0.06 mm CG3(gIII) mean 1.72 +/− SD 0.06 mm EG1(gIV) mean 2.11 +/− SD 0.0.04 mm EG2(gV) mean 2.16 +/− SD 0.06 mm **%** n.a. ***p* < 0.001** between CG1/CG2/CG3 groups and both EG1/Eg2 groups ***p*** > between EG1 and EG2
10. Seifi et al. [35], 2003,	WR (rats), 8-week-old male, (24), 21 days	Prostaglandin E2 (PGE2) alone and with calcium gluconate (Ca), submucosally injected at the mesiobuccal mucosa of the right first molars	60 g, mesialization of right maxillary first molar to incisors	Administration frequency—twice: on days 0 and 7 EG1(g3): OTM + 0.1 mL of 1 mg/mL PGE2 dissolved in 1 per cent lidocaine EG2(g4): OTM + PGE2 same dosage + 10% Ca (200 mg/kg) injected intra-peritoneally PCG(g2—right side): OTM + 0.1 mL distilled water NCG (g1—left side): no OTM + no intervention	**amount of OTM** PCG mean 0.2162 +/− SD 0.0995 and range 0.14–0.45 mm EG1 mean 0.4700 +/− SD 0.2799 and range 0.20–0.91 mm EG2 mean 0.4012 +/− SD 0.1007 and range 0.29–0.57 mm **%** n.a. ***p* < 0.05** for EG1 compared to the control group
11. Leiker et al. [36], 1995, EG+CG	SDR (rats), 8-week-old male, (132), G1: 2 week/G2: 4 week	Prostaglandin E2 (PGE2), injected in the mesiolingual gingiva of each maxillary first molar	60 g, mesialization of first maxillary molar to incisors	G1a: single (1) injection G1b: 2 injections once a week (total 2) G2a: single (1) injection G2b: 4 injections once a week (total 4) EG: OTM + subgroups were further divided into four concentration subgroups (0.1, 1.0, 5.0, and 10.0 µg of PGE2) CG: OTM + PBS	**amount of OTM**—n.a. (values presented in the article as an image) **%** n.a. ***p* < 0.05** in all PGE2 groups compared to their respective non-PGE2 groups—overall at ***p*** < 0.0001
12. Yamasaki et al. [37], 1982, S-M	Macaca Fukasata (monkey), 5-year-old female, (2), Exp1: 18 days, Exp2: 28 days	Prostaglandin E1 and prostaglandin E2 (PGE1 No. G 511* and PGE2 No. G 512* *Ono Company, Ltd., Osaka, Japan—dissolved in saline at a concentration of 160/ug/mL.), injected in the submucosal area of the distal side of the upper right canine	100 g, distalization of upper right maxillary canine to second premolar	Exp1: days 0, 1, 5, 9, 12 and 15 EG: OTM + 0.25 mL of PGE2-CD (40 µg) CG: OTM + PBS Exp2: rate not specified; Eside and Cside reversed on day 14 EG: OTM + PGE1-CD CG: OTM + PBS	**amount of OTM** Exp1 0.5 mm TM day 1, control 0.5 mm 1.3 mm TM day 5, control 0.5 mm 2.5 mm TM day 15, control 1.2 mm Exp2 “almost double amount” of TM compared to the control side for the first two weeks “TM results were reversed” in the second 2-week period, and ”by the third week … both sides had the same amount of movement” switched sides on day 14 “caused almost double the rate of tooth movement compared to the vehicle-injected side” **%** n.a. *p*-value n.a.
13. Peng et al. [44], 2021, EG + CG	SDR (rats), 6- to 8-week-old male, (80), 14 days	Recombinant human insulin-like growth factor-1 (Pepro-Tech, Rocky Hill, NJ, USA), (injection) in the lateral buccal mucosa of the left maxillary first molar	50 g, left first upper molar mesialization to incisors	Every 2 days EG: OTM + 400 ng rhIGF-1 (Pepro-Tech, USA) CG: OTM + PBS	**amount of OTM** CG 0.040 ± 0.040 mm EG 0.076 ± 0.045 mm day 1 CG 0.164 ± 0.010 mm EG 0.222 ± 0.010 mm day 4 CG 0.267 ± 0.013 mm EG 0.369 ± 0.007 mm day 7 CG 0.339 ± 0.015 mm EG 0.481 ± 0.008 mm day 10 CG 0.410 ± 0.014 mm EG 0.581 ± 0.009 mm day 14 **%** n.a. ***p* < 0.01** from day 4 to day 14
14. Seifi et al. [45], 2013, EG + CG	WR (rats), 4-month-old male, (50), 21 days	Basic fibroblast growth factor [(bFGF)—Royan Institute] (cytokine), injection into the buccal vestibular mucosa next to the mesial root of the first molar	60 g, mesialization of right first upper molar to incisor	Once, immediately after establishing the OTM model EG(A): OTM + 0.02 cc of 10 ng bFGF (Royan Institute) EG(B): OTM + 0.02 cc of 100 ng bFGF EG(C): OTM + 0.02 cc of 1000 ng bFGF PCG(D): OTM + PBS NCG(E): no OTM + no intervention	**amount of OTM** EG(A) mean value 0.5333 mm EG(B) mean value 0.6633 mm EG(C) mean value 0.7700 mm PCG(D) mean value 0.2550 mm NCG(E) mean value 0.0217 mm **%** n.a. ***p* < 0.05** in all EG compared to PCG ***p* < 0.05** in EG(C) compared to EG(A)
15. Alves et al. [42], 2009, EG + CG	HR (rats), male, (96), 21 days	Epidermal growth factor (EGF-liposomes), injected in the mucosa adjacent to the appliance of the left side	20 g, mesialization of left maxillary first molars to incisors	Once, immediately applied after establishing the OTM model, then daily PBS EG1(G2): OTM + empty liposomes in 10 μL of PBS solution EG2(G3): OTM + vesicles containing 0.5 ng/μL of EGF with ~100 nm of diameter in 10 μL of PBS solution (20 ng of EGF) EG3(G4): OTM + 20 ng of EGF–liposomes in 10 μL of PBS solution CG(G1): OTM + PBS	**amount of OTM**—n.a. (values presented in the article as an image) **%** n.a. ***p* < 0.05** in all four periods analyzed, animals that received an injection of EGF–liposome solution showed increased tooth movement (*p* < 0.05) as compared to the three groups
16. Saddi et al. [43], 2008, EG + CG	HR (rats), male, (32), 5 days	Epidermal growth factor (EGF—SOLUBLE), injected into the region of the root furcation of the left first molar	Insertion of elastic band between first and second molars, mesialization of the left maxillary first molar	Once, immediately applied after establishing the OTM model EG1(GI): OTM + 20 ng EGFliposomes in 10 uL PBS solution CG1(GII): OTM + liposomes in 10 uL PBS solution EG2(GIII): OTM + 20 ng EGF in 10 uL PBS solution CG2(GIV): OTM + 10 uL PBS solution	**amount of OTM** EG1(GI) 0.55 mm CG1(GII) 0.26 mm EG2(GIII) 0.42 mm CG2(GIV) 0.26 mm **%** n.a. ***p* < 0.05** for both experimental groups compared to control
17. Brooks et al. [46], 2011, EG + CG	Mi (mice), 10-week-old male, (48), 6 days	Macrophage colony-stimulating factor (M-CSF—*Calbiochem*, *Gibbstown*, *N*))—an early osteoclast recruitment/differentiation factor, injected sub-periosteally into the distopalatal root of the right maxillary first molar	12 (cN), mesialization of right maxillary first molars to incisors	Once, immediately applied after establishing the OTM model EG1(low): OTM + 0.1 mg/kg doses of recombinant mouse M-CSF (Calbiochem, Gibbstown, NJ, USA) EG2(high): OTM + 1 mg/kg doses of recombinant mouse M-CSF (Calbiochem, Gibbstown, NJ, USA) PCG: OTM + PBS NCG: no OTM	**amount of OTM** PCG mean value 72 +/− 8.4 um day 2 (lag after tipping phase) PCG mean value 193 +/− 5.7 um day 6 (linear movement phase) NCG mean value 0 +/− 2.0 um day 2 NCG mean value 0 +/− 1.0 um day 6 EG1 (low) mean value 64 +/− 8.9 um day 2 EG1 (low) mean value 220 +/− 18.3 um day 6 EG2 (high) mean value 68 +/− 8.4 um day 2 EG2 (high) mean value 200 +/− 14.1 um day 6 % 14% increase after 6 days of treatment ***p* < 0.05** on day 6 for EG1(low dose) compared to PCG ***p* > 0.05** on day 6 for EG2(high dose) compared to PCG
18. Jung et al. [53], 2018, EG + CG	Beagles (dogs), 18- to 24-month-old male, (8), 4 weeks	Exogenous thyroxine, transmucosal administration (via tablets bonded on the orthodontic appliance)	100 (cN), mesialization of upper second premolars to canine and distalization of lower second premolars to fourth premolar and first molar	Starting from the application of the OTM model, tablets changed once a week EG: OTM + one dose of Thyroxine with a rate of dissolution of 18.69% after 24 h. CG: OTM + no intervention	**amount of OTM** EG—maxilla mean value 0.19 +/− SD 0.08 mm/week CG—maxilla mean value 0.18 +/− SD 0.10 mm/week EG—mandible mean value 0.20 +/− SD 0.08 mm/week CG—mandible mean value 0.16 +/− SD 0.07 mm/week **%** n.a. ***p* > 0.05**
19. Hashimoto et al. [49], 2001, EG + CG	WR (rats), 5-week-old male, (88), 10 days	Osteocalcin (OC—a major noncollagenous bone matrix protein—purified rat OC), injected in the palatal subperiosteum adjacent to the furcation of the maxillary right first molar	30 g, mesialization of the right maxillary first molar to incisor	The first administration was given on the day that the orthodontic appliance was inserted and repeated daily until day 9. EG1: OTM + 0.1 μg OC EG2: OTM + 1 μg OC EG3: OTM + 10 μg OC CG1: OTM + PBS CG2: OTM + albumin CG3: OTM + no intervention	**amount of OTM**—n.a. (values presented in the article as an image) % EG1 147% EG2 152% EG3 121% TM Compared to CG1 ***p* < 0.05** EG1 and EG2 compared to CG1 and CG3 on day 10
20. Kobayashi et al. [50], 1998, EG + CG	WR (rats), 5-week-old male, (33), 4 days	Osteocalcin (OC—a major noncollagenous bone matrix protein— purified rat OC *described in this article*), injected into the submucosal palatal area corresponding to the root furcation of the maxillary right first molar	Insertion of elastic band between first and second molars, mesialization of the right maxillary first molar	Injection was repeated once a day from day 0 to 3 EG1: OTM + 0.01 µg purified osteocalcin/20 µL PBS EG2: OTM + 0.1 µg purified osteocalcin/20 µL PBS EG3: OTM + 1 µg purified osteocalcin/20 µL PBS EG4: OTM + 10 µg purified osteocalcin/20 µL PBS CG: OTM + PBS	**amount of OTM** maximal amount of OTM observed in EG3 (1 µg OC) **%** n.a. ***p* < 0.05** for EG3 (1ug OC) compared to CG on all 4 days ***p* < 0.05** for EG4 (10 µg OC) starting on day 3 ***p* < 0.05** for EG2,3,4 on day 4
21. Lu et al. [54], 2019, S-M	WR (rats), 6-week-old male, (48), 14 days	Sclerostin protein (R&D systems, Minneapolis, MN, USA) carried by PECE hydrogel, local injection at the compression side in the alveolar bone, approximately 4mm mesial from the maxillary first molar	50 g, randomly left or right first upper molar mesialization to incisors	Once, immediately after establishing the OTM model ES1: OTM + 0.1 mL 0.8 μg/kg sclerostin protein carried by hydrogel (R&D systems, MN, USA) ES2: OTM + 0.1 mL 4 μg/kg same substance ES3: OTM + 0.1 mL 20 μg/kg same substance CS: OTM + PBS	**amount of OTM** ES1: mean value 0.58 ± SD 0.07 mm ES2: mean value 0.65 ± SD 0.06 mm ES3: mean value 0.72 ± SD 0.04 mm **%** n.a. ***p* < 0.01** for ES2,ES3 compared to ES1 and CS
22. Chung et al. [55], 2007, EG + CG/S-M	SDR (rats), 6- to 7-week-old male, (25), 7 days	A specific EP4 agonist (ONO-AE1-329), a drug that binds to the EP4 receptor to mimic the actions of ligand binding, injected locally into the distopalatal mucosa region of the right first molar under diethyl ether anesthesia	6,5 (cN), reciprocal force: mesiodistal movement of the right maxillary first, second and third molar	The first injection was made on day 0; then injections were given twice a day at 7-h intervals starting from day 3 until the rats were killed on the evening of day 7 (total of 10 injections). CG1 (EA—EP4 agonist): no OTM + 250 µL ONO-AE1-329 CG2 (V—vehicle): no OTM + vehicle EG1 (TMEA—tooth movement and EP4 agonist): OTM + 250 µL ONO-AE1-329 EG2 (TMV—tooth movement and vehicle): OTM + vehicle	**amount of OTM** n.a. **%**—n.a. (values presented in the article as an image) ***p* < 0.05** for EG3 compared to EG4
23. Bolat et al. [51], 2020, EG + CG	WAR (rats), 6- to 8-week-old male, (51), 18 days	Vitamin C and Vitamin E, local injections were performed with a microsyringe into the periodontal area of the maxillary first molar	50 g, mesialization of maxillary first molar to incisors	Administration every 3 days EG1: OTM + locally injected Vitamin C 20 μL EG2: OTM + locally injected Vitamin E 20 μL EG3: OTM + intraperitoneal 150 mg/kg vitamin C EG4: OTM + intraperitoneal 150 mg/kg vitamin E CG: OTM + no intervention	**amount of OTM** In this experimental study, the application of systemic or local vitamin C and E did not affect the orthodontic tooth movement rate. **%** n.a. ***p* < 0.05** (The osteoblastic activity was considerably raised in all the vitamin groups. Additionally, the groups treated with systemic vitamins showed a significant increase in the quantity of collagen fibers on the tension side compared to the control group using the appliance.)
24. TakanoYamamoto et al. [38], 1992, EG + CG	WR (rats), young 7-week-old male (10) and adult 28-week-old male (30) (total 40), 21 days	1,25(OH)2D3 –Calcitriol (Sunstar Co., Osaka, Japan Roche Co., Tokyo, Japan)—active form of vitamin D, injected locally into the submucosal palatal area of the root bifurcation of the right first molar	5 to 20 g, buccal movement of the first upper molar	Every 3 days (young)EG: OTM + 1,25-(OH)2D3 20 μL of 10^−10^ mol/L (young)CG: OTM + PBS (adult)NCG1: no OTM + PBS (adult)PCG1: no OTM + 1,25-(OH)2D3 20 μL of 10^−10^ mol/L (adult)PCG2: OTM + PBS (adult)EG4: OTM + 1,25-(OH)2D3 20 μL of 10^−10^ mol/L (adult)EG5: OTM + 1,25-(OH)2D3 20 μL of 10^−8^ mol/L	**amount of OTM**— young—0.5 mm adult—1.2 mm **%** for (young)EG—increased to 126% of that in PBS-injected control rats on day 20 for (adult)EG4—increased to 245% compared to PBS by the end of the experiment for (adult)EG5—increased to 154% compared to PBS by the end of the experiment ***p* < 0.05** (young)EG—starting from day 15 to day 20 as compared with the rats which were injected with PBS
25. Collins et al. [28], 1988, EG + CG/S-M	Cats, young adult, (10), 22 days	Calcitriol—(1,25-dihydroxycholecalciferol—a vitamin D metabolite), intraligamentary injection into the distal portion of the periodontal ligament of canine teeth	80 g, distalization of upper left canines to the third premolars	The first injection was made immediately after establishing the OTM model, then repeated once a week EG: OTM + 0.1 mL of DMSO containing 50 µg/mL of 1,25D CG: OTM + 0.1 mL of DMSO only	**amount of OTM**—n.a. (values presented in the article as an image) **%** EG teeth moved 60% further than their matched control after 21 days (also divided by weeks) ***p* < 0.05**
26. Ma et al. [57], 2020, EG + CG	SDR (rats), 8-week-old female, (64), 14 days	Asperosaponin VI (ASA VI—chemical constituent isolated from Dipsacus asper Wall), injected into buccal submucoperiosteal of first upper molars	40 g, mesialization of bilateral upper first molars to incisors	Once a day, every day during the orthodontic tooth movement period EG: OTM + 10 mg/kg ASA VI solution CG: OTM + PBS	**amount of OTM**—n.a. (values presented in the article as an image) % EG—approximately 1.2 times greater at day 3, 1.44 times greater at day 7, and 1.54 times greater at day 14. A significant difference was found between the ASA VI group and the control group on day 7 and day 14 ***p* < 0.05**
27. Kavoli et al., [56], 2017, EG + CG	WR (rats), 4 months old, (28), 21 days	Carrageenan (CGN— carrageenan 1% Sigma-Aldrich, St. Louis, MO, USA), a common food additive, injected into the mucosa of the buccal vestibule adjacent to the mesial root of the left first molar	20 g, mesialization of left upper first molars to incisors	Once, immediately applied after establishing the OTM model EG: OTM + 40 mL carrageenan 1% (Sigma-Aldrich, St. Louis, MO, USA) CG: OTM + PBS	**amount of OTM**—n.a. (values presented in the article as an image) **%** injection of carrageenan can speed up tooth movement by about 58% and increase the presence of osteoclasts by 40%, after 21 days; local injection of carrageenan is a method capable of accelerating orthodontic tooth movement by about 1.6-fold ***p* = 0.053**
28. Xiao et al. [58], 2015, EG + CG	SDR (rats), 6- to 8-week-old male, (150), 30 days	Aqueous extract of S. miltiorrhiza (ESM—Danshensu—content > 98%; Nanjing Zelang pharmaceutical Technology Co., Ltd., Nanjing, China; No.: ZL201104162;), injected into the buccal vestibular mucosa of first molar of left maxilla	40 g, mesialization of left upper first molars to incisors	Once a day, every day CG: OTM + 0.5 mL/kg PBS EG1: OTM + 0.5 mL/kg ESM, which was equivalent to 0.75 g/kg of crude drugs) EG2: OTM + 0.5 mL/kg Danshensu, which as equivalent to 250 mg/kg of body weight EG3: OTM + 0.5 mL/kg Danshensu, which as equivalent to 500 mg/kg of body weight EG4: OTM + 0.5 mL/kg Danshensu, which as equivalent to 750 mg/kg of body weight	**amount of OTM**—n.a. (values presented in the article as an image) **%** n.a. ***p***- from day 10 EG1 ***p* < 0.01**, from day 20 also EG2, EG3 and EG4 ***p* < 0.05.**

EG = experimental group; CG = control group (PCG—positive control group with appliance, NCG—no appliance); DMSO—Dimethyl sulfoxide; PBS—Phosphate Buffer Saline; PDL—periodontal ligament; OTM/TM = orthodontic tooth movement = orthodontic appliance was set and activated; S-M = split mouth design; EG + CG = experimental group(s) + control group(s).

**Table 6:** Pharmacokinetics of the investigated pharmacological substances.

	PGE1	PGE2	Calcitriol	PTH	Exogenous Thyroxine	Vitamin C
**Synonyms**	Alprostadil, Prostaglandin E1	Dinoprostone, Prostaglandin E2	1-alpha-25-Dihydroxyvitamin D3	Parathormone, Parathyroid hormone	L-T4, Levothyroxin, Thyroxine	Ascorbate, Ascorbic acid
**Chemical formula**	C_20_H_34_O_5_	C_20_H_32_O_5_	C_27_H_44_O_3_	C_408_H_674_N_126_O_126_S_2_	C_15_H_11_I_4_NO_4_	C_6_H_8_O_6_
**Type and weight**	Small molecule, Average: 354.487 Monoisotopic: 354.240624195	Small molecule, Average: 352.4651 Monoisotopic: 352.224974134	Small molecule, Average: 416.6365 Monoisotopic: 416.329045274	Single-chain polypeptide (mature protein), Protein average weight: 9420.0 Da	Small molecule, Average: 776.87 Monoisotopic: 776.686681525	Small molecule, Average: 176.1241 Monoisotopic: 176.032087988
**Background**	Alprostadil is a synthetic form of prostaglandin E1, a potent vasodilator for treating erectile dysfunction by promoting smooth muscle relaxation; it can also be used in neonatal patients with congenital heart defects, causing vasodilation and increased blood flow.	Significant impact on labor; It has stimulating effects on osteoblasts to secrete substances that promote bone resorption by osteoclasts; as a prescription substance, it is used as a vaginal capsule to prepare/induce labor.	Calcitriol is a potent vitamin D metabolite produced through UV light exposure; as a prescription substance it is used for treating secondary hyperparathyroidism, metabolic bone disease, hypocalcemia, osteoporosis, and mild to moderate plaque psoriasis in adults, administered orally and intravenously.	Used to treat hypocalcemia resulting from hypoparathyroidism, PTH is an analog of human parathyroid hormone.	Oral levothyroxine is a synthetic hormone that maintains normal T4 levels in hypothyroidism. Thyroid hormones, T4 and T3, have a potency of approximately 1:4 and have a strong effect on the cardiac system, making their status closely linked to heart rate, cardiac output, and systemic vascular resistance.	Vitamin C is a vitamin that is used to treat vitamin C deficiency, scurvy, prolonged wound and bone healing, urinary acidity, and as an overall antioxidant. It is also thought function as a reducing agent and coenzyme in various metabolic pathways and is considered an antioxidant.
**Mechanism of action**	Alprostadil is a smooth muscle relaxant used in neonatal patients with ductus arteriosus patency to prevent or reverse functional closure of the ductus arteriosus, increasing blood flow. In adult men it relaxes the trabecular smooth muscle of the corpora cavernosa and cavernosal arteries, leading to swelling, elongation, and rigidity due to the corporal veno-occlusive mechanism.	Dinoprostone, when administered intravaginally, stimulates the myometrium of the gravid uterus, similar to labor contractions, resulting in the evacuation of conception products. Its exact mechanism is unknown, but it may regulate calcium transport and intracellular concentrations. It also produces local cervical effects, possibly due to collagen degradation.	Calcitriol, as a psoriasis treatment, has antiproliferative effects on keratinocytes and promotes epidermal cell differentiation. Its anticarcinogenic action is linked to cellular vitamin D receptor (VDR) levels, which activate or suppress target gene transcription. VDRs, expressed in monocytes but induced after T and B cell activation, also mediate calcitriol’s immunomodulating action. It also enhances the activity of certain vitamin D-receptor positive immune cells and sensitivity to cytokines generated by immune cells.	rhPTH’s biological actions are mediated by binding to two high-affinity cell-surface receptors for its N-terminal and C-terminal regions, which are essential for normal bone metabolism. The N-terminal region is responsible for parathyroid hormone’s bone building effects, while the C-terminal region regulates N-terminal fragment activity, demonstrating its antiresorptive activity.	Thyroid hormone boosts body cell metabolism, aiding in growth and development of tissues like bones and brain. In adults, it maintains brain function, food metabolism, and body temperature.	Ascorbic acid is essential for collagen formation and tissue repair in humans, and is reversibly oxidized to dehydroascorbic acid. It is involved in tyrosine metabolism, folic acid conversion, carbohydrate metabolism, iron metabolism, resistance to infections, and cellular respiration.
**Absorption**	Patients who received 20 μg of alprostadil intracavernously showed increased systemic plasma concentrations with a tmax and AUC of 4.8 min and 173 pg⋅min/mL, respectively. The absolute bioavailability of alprostadil from systemic exposure was estimated to be around 98% compared to a short-term intravenous infusion.	Absorbed at 0.3 mg per hour for 12 h while the system is in place.	Intestinally absorbed, has a mean serum concentration of 60.0 ± 4.4 pg/mL at 2 h. Its peak plasma concentrations are reached within 3 to 6 h, with an oral bioavailability of 70.6 ± 5.8% in healthy patients.	For dosages of 100 micrograms, the absolute bioavailability following subcutaneous abdominal injection is 55%.	T4 absorption from the gastrointestinal tract is 40% to 80%, with most absorbed from the jejunum and upper ileum. Absorption is influenced by fasting, malabsorption syndromes, certain foods, age, and drugs like bile acid sequestrants and minerals.	70% to 90%
**Volume of distribution**	n.a.	n.a.	Calcitriol distribution volume was 0.49 ± 0.14 L/kg in healthy male individuals after intravenous treatment. There is evidence that calcitriol is transmitted into human milk at low quantities (2.2 ± 0.1 pg/mL) in mothers. Calcitriol from maternal circulation may enter fetal circulation.	Following intravenous injection, the volume of distribution at steady-state is roughly 5.4 L with an interpatient range of roughly 40%.	n.a.	n.a.
**Protein binding**	Mostly to albumin (81%) and, somewhat less for alpha-globulin IV-4 fraction (55%)	73%, to albumin	99.9%, to an alpha-globulin vitamin D binding protein	n.a.	99% bound to plasma proteins (thyroxine-binding globulin, thyroxine-binding prealbumin and albumin)	25%
**Metabolism**	Rapidly metabolized with a smaller portion absorbed in the systemic circulation and distributed throughout the body, except for the central nervous system. 60-90% of the circulating substance may be metabolized in the lungs.	PGE2’s rapid metabolism occurs primarily in local tissues, with systemic absorption cleared mainly in maternal lungs and subsequently in the liver and kidneys.	Calcitriol metabolism involves two pathways: 24-hydroxylase activity in kidneys and target tissues like intestines, producing calcitroic acid, and stepwise hydroxylation of carbon-26 and carbon-23, resulting in 1a,25R(OH)2-26,23S-lactone D3, the major human metabolite.	PTH is primarily metabolized in the liver. Amino terminal fragments are metabolized in the liver, while carboxyl terminal groups are transported to the kidney for metabolism. Only 30% of circulating hormone is unfragmented.	70% of secreted T4 is deiodinated to T3 and reverse triiodothyronine, with 80% circulating T3 derived from peripheral T4; liver, kidney, and other tissues are major sites of degradation.	Hepatic; ascorbic acid undergoes reversible oxidation to dehydroascorbic acid, two active forms in body fluids, and some is metabolized to inactive compounds like ascorbic acid-2-sulfate and oxalic acid.
**Route of elimination**	Metabolites are primarily excreted by the kidney within 24 h of administration, with 88% and 12% excreted through urine and feces over 72 h. Alprostadil and its metabolites are not retained in tissues.	Kidneys	27% and 7% of radioactivity appears in feces and urine, respectively within 24 h; calcitriol undergoes enterohepatic recycling and biliary excretion.	The kidney filters carboxy-terminal fragments, which are then fragmented during tubular reuptake.	Thyroid hormones are primarily eliminated by kidneys, with some remaining in the colon and 20% in the stool, with urinary excretion decreasing with age.	n.a.
**Half-life**	5–10 min	<5 min	5–8 h	1.5 h	6–7 days	16 days
**Clearance**	Total body clearance 115 L/min after intravenous administration (20 μg)	n.a.	The metabolic clearance rate in healthy males was 23.5 ± 4.34 mL/min, while in male patients with uremia, it was 10.1 ± 1.35 mL/min, and in pediatric patients, 15.3 mL/hr/kg.	n.a.	n.a.	n.a.
**Toxicity**	Oral LD_50_ in mice and rats is 186 mg/kg and 228 mg/kg, respectively.	Oral, mouse: LD_50_ = 750 mg/kg; Oral, rat: LD50 = 500 mg/kg.	LD_50_ (oral, rat) = 620 μg/kg; LD50 (intraperitoneal, rat) > 5 mg/kg	n.a.	LD_50_ = 20 mg/kg (orally in rat)	n.a.
**State**	Solid	Solid	Solid	Solid	Solid	Solid
**Melting point (°C)**	115–116 °C	67 °C	113–114 °C	n.a.	235.5 °C	191 °C
**Water solubility**	26.7 mg/L	58.1 mg/L	Insoluble	n.a.	0.105 mg/mL	4E + 005 mg/L (at 40 °C)
**logP**	3.20	2.82	5	n.a.	4	−1.85

## Data Availability

Data sharing not applicable. No new data were created or analyzed in this study. Data sharing is not applicable to this article.
